# The use of body condition and haematology to detect widespread threatening processes in sleepy lizards (*Tiliqua rugosa*) in two agricultural environments

**DOI:** 10.1098/rsos.140257

**Published:** 2014-12-24

**Authors:** Anita K. Smyth, Elizabeth Smee, Stephanie S. Godfrey, Mathew Crowther, David Phalen

**Affiliations:** 1 CSIRO, PMB 2, Glen Osmond, South Australia 5064, Australia; 2 Future Farm Industries Cooperative Research Centre, The University of Western Australia, 35 Stirling Highway, Crawley, Western Australia 6009, Australia; 3 TERN Eco-informatics Facility, The University of Adelaide, North Terrace, Adelaide, South Australia 5005, Australia; 4 School of Biological Sciences, The University of Sydney, Sydney, New South Wales 2006, Australia; 5 School of Biological Sciences, Flinders University, GPO 2010, Adelaide, South Australia 5000, Australia; 6 School of Veterinary and Life Sciences, Murdoch University, 90 South Street, Murdoch, Western Australia 6163, Australia; 7 Faculty of Veterinary Science, The University of Sydney, Sydney, New South Wales 2570, Australia

**Keywords:** anaemia, haemolysis, farm chemicals, haematology

## Abstract

Agricultural practices, including habitat alteration and application of agricultural chemicals, can impact wildlife resulting in their decline. Determining which of these practices are contributing to declines is essential if the declines are to be reversed. In this study, the health of two geographically separated sleepy lizard (*Tiliqua rugosa*) populations was compared between a rangeland environment and cropping environment using linear body size index (LBSI) and haematology. Animals in the cropping site were smaller, suggesting genetic differences as the result of geographical isolation. The animals in the cropping site had a lower LBSI and many were experiencing a regenerative anaemia. The anaemia was postulated to be the cause of the low LBSI. The anaemia appeared to be the result of haemolysis and was likely to be caused by exposure to agricultural chemicals applied in the cropping site but not the rangeland site. Elevated white blood cell counts in lizards in the rangeland site suggested that they were experiencing an inflammatory disease of possible ecological significance. Together, these results demonstrate the value of combining physical and haematological parameters when studying the impact of agricultural practices on wildlife. They also show that reptiles may be useful as sentinel species for livestock and humans.

## 2. Introduction

Agricultural practices can significantly impact wildlife. Much is known about habitat fragmentation of agricultural environments and how it negatively affects animal habitats, vertebrate populations [1] and its secondary effects on health profiles of free-ranging individuals [2]. Less is known about the impact of the widespread and commonly used agricultural chemicals on wildlife living in these environments [3,4]. Individually or in combination, changes in the ecosystem, exposure to environmental chemicals and other threatening processes not directly related to agriculture can result in population declines and in some cases regional or widespread extinctions [5,6]. Determining what factor or combinations of factors are causing wildlife declines in agricultural areas is essential if these declines are to be reversed.

A species' decline may reflect threatening processes that are having wider ecosystem ramifications [7]. As canaries were recognized by miners as being more susceptible to toxic gases than humans, the value of domestic animals and wildlife as sentinel species for environmental toxins has grown [8–10]. Thus, declines in one or more wildlife species in agricultural areas could be indicative of exposure to levels of chemicals that are approaching concentrations that could have widespread ecological impacts including impacts on the health of domestic animals and humans.

Despite reports of declines in reptile assemblages [6], abnormal biochemical profiles in reptiles from agricultural areas [3,4], direct impact of some insecticides on reptiles and global calls for more attention [11,12], only a limited number of studies have examined the value of terrestrial reptiles as sentinel species in agricultural environments. Reptiles have the potential to be important environmental sentinels as they are generally sedentary, are terrestrial and are often abundant in agricultural and adjacent environments [13]. Additionally, they can be herbivores, omnivores or carnivores, can be exposed to chemicals by multiple routes (inhalation, ingestion and direct skin contact), and because some species can live for many decades, impacts of cumulative toxin exposure may be observed [3,4].

The sleepy lizard (*Tiliqua rugosa*) has the potential to be an important sentinel of environmental health. This medium-sized lizard occurs throughout southern Australia, has flexible habitat requirements and lives in diverse vegetation types modified by livestock grazing or cereal cultivation [14]. It is a long-lived (20–50 years), ectothermic, scincid lizard that is terrestrial and manually captured with ease. Adults are monogamous and have stable home ranges (200 m^2^–1 km^2^) that overlap during the breeding season (September–December) [15]. Young are born mostly annually (assuming pairings lead to breeding success) ([16] and references therein) and remain within the same home range as their mothers during the first year [17]. They emerge from winter ‘hibernation’ in late August, bask until ground temperatures reach about 23^°^C and then feed mostly on annual forbs, seeds and occasionally on carrion [18,19]. Little is known about the impact of agricultural practices on the health of this species.

Haematology is minimally invasive and can be used to monitor the health status of reptiles [5]. With other measures of health, it is a powerful tool that can assist in identifying possible causes of species declines. Specifically, changes in red blood cell (RBC) parameters can provide evidence of blood loss, RBC destruction and the rate of RBC regeneration that can be impacted by infectious agents, toxins, nutritional status, stress and chronic disease. Changes in blood protein concentrations can reflect blood loss, chronic disease and nutritional status, while changes in the leucogram can provide evidence of intoxication, acute and chronic infectious diseases, and have been suggested to reflect acute and chronic stress. Additionally, examination of blood can also be used to detect the prevalence and intensity of infection of blood parasites, and some bacteria and viruses [20,21].

Our study explores the health of two free-ranging (wild) populations of the sleepy lizard from unimproved rangelands and the nearby ‘intensively managed’, fragmented landscapes of southern Australia's cereal croplands using physical and haematological measures of health. We propose that cropland individuals will display ‘secondary effects’ of cultivation based on compromised health measures (body condition, blood profiles and ectoparasite load) compared to rangeland individuals which exhibit a pseudobasal range of health measures.

## 3. Material and methods

### 3.1 Study sites

**Figure 1.**
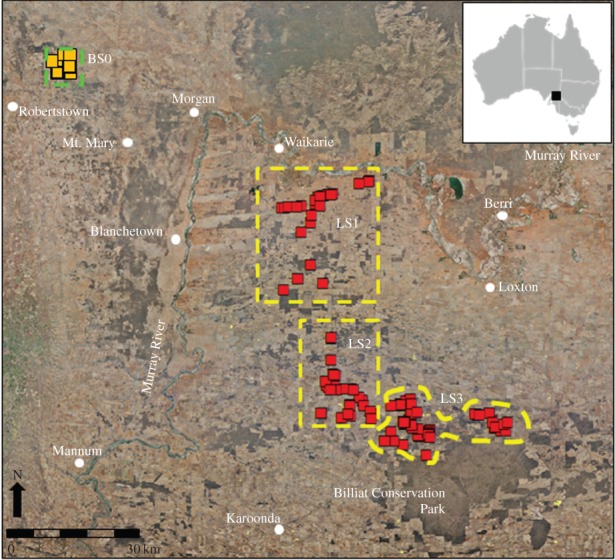
Location of the Baseline study site (BS0: orange) in the rangelands and Severe study sites (red) in the cereal cropland landscapes (LS1, LS2 and LS3) in the SAMM region of southern Australia (north of Murray River—rangelands, south of Murray River—cropland).

This study took place in the South Australian Murray Mallee (SAMM) region [22]. Adult *T. rugosa* were captured for sampling at one rangeland (Baseline) site (BS0–120 km^2^) near Mt. Mary (139^°^21′ E, 33^°^55′ S) and adults and juveniles at three severely modified (Severe) landscape-scaled sites (LS1, LS2 and LS3) over a large area (68×84 km or 571 200 ha) across the croplands. The Baseline and the Severe sites are separated by the Murray River (figure 1). In the absence of physiological standards for wild *T. rugosa*, animals sampled from the Baseline site were considered to be exposed to fewer agricultural chemicals (see Discussion) and less habitat alteration, and therefore provided a relative basal range of physiological health. Conversely, animals sampled from the Severe sites were exposed to the cumulative effects of habitat alteration and exposure to numerous agricultural chemicals associated with cereal cultivation. These sites varied in size (LS1–730 km^2^, LS2–625 km^2^, LS3–540 km^2^) and were selected based on the spatial configuration of lizard habitat such that each site contained combinations of revegetated saltbush and roadside verge and patches of remnant mallee woodland. All Severe sites captured the widespread decline in structural connectivity of lizard habitat at the landscape scale (derived using the ARCGIS 10 spatial analysis tool). The Baseline and Severe sites were sampled in November 2010 and during September and November 2010, respectively. Mean monthly rainfall (March 2010 to February 2011) was 37.6 mm (total=451.6 mm) at the Baseline site and 49.9 mm (total=598.2 mm) for the Severe sites. Monthly maximum temperature varied from 12.7^°^C (August) to 31.7^°^C (January) in the Baseline site and 16.1^°^C (August) to 32.2^°^C (January) in Severe sites.

### 3.2 Animal sampling

We used a single treatment (fixed, two levels: Baseline habitat alteration, *n*=30; Severe habitat alteration, *n*=30) and one covariate for body condition (two measures: linear body size index (LBSI) or ectoparasite load, where *n*=30 for each) to investigate lizard health. All animals were grouped into two juveniles (snout vent length, SVL<200 mm) and adults which included two sub-adults (SVL>200 mm) [23] but not by sex because of unreliable sex determination using eversion of hemipenes [23]. The effect of brumation on haematological data in the Severe sites was assessed using cumulative days since the first day of sampling (CUMDAYS) [24].

Lizards were surveyed in both types of sites by employing a single reptile visual encounter survey published for another study [22]. Surveys were undertaken multiple times in the Baseline site and once in woodland remnants and revegetated saltbush within the Severe sites. At the Baseline site, blood smears (coverslip–slide method) were collected via toe-clipping and prepared using immediate duplicate blood smears [25–27]. At the Severe sites, animals were microchipped and blood taken from the caudal tail vein [22]. Immediate duplicate blood smears were prepared and later stained with a Wrights–Giesma stain (Sigma-Aldrich). To prevent the impact of leucocyte trafficking on the blood cell counts [28], blood samples were collected immediately, with the exception of two animals from the Severe site, where a 10–15 min delay in blood collection occurred.

### 3.3 Haematological processing

Heparinized blood from the Severe sites was transferred to a capillary tube and the percentage of the blood that was composed of RBCs (packed cell volume (PCV)) was determined using a microcentrifuge (*n*=85). PCV was not determined for Baseline animals.

Differential counts for all lizards were determined by counting 100 white blood cells (WBCs) with the 100× objective and recording the percentage of heterophils, lymphocytes, monocytes and other granulocytes [29]. Ten random fields of view with even distributions of erythrocytes were selected for viewing. The edges of the clover slip were avoided to minimize inaccuracies caused by clumped cells. The other granulocyte count included cells that matched the description of basophils seen in other reptile species [29], another cell type that contained clear round vacuoles that appeared to have degranulated, and large round cells with fine basophilic granules. The total number of heterophils from all animals in the control site was estimated by counting the number of heterophils present in each of five fields using the 40× objective of the microscope in an area of the blood smear where the RBCs first became overlapping. The number of cells per field was then averaged and the average was multiplied by 2000 to give an estimated number of heterophils per microlitre [29]. The fraction of heterophils was then divided into the estimated total heterophil count to give the total WBC count and this was used to calculate the total monocyte, lymphocyte and other granulocyte total counts. The percentage of polychromatophilic RBCs was determined by counting the number of polychromatophilic RBCs per 200 RBCs.

Total WBC counts were not done for samples collected from the Severe sites because the estimated WBC count is linked to the number of RBCs. Thus, varying degrees of anaemia add considerable uncertainty to the actual count. Additionally, the quality of the blood smear made from blood with a low PCV is often poor, resulting in an uneven distribution of cells adding additional uncertainty to the WBC estimate. Automated total WBC counts were preferable but due to unforeseen circumstances were not possible for this study.

### 3.4 Body size

Body size was assessed using the unity of LBSI. LBSI was calculated from the residuals of a ranged major axis (RMA) regression between log-transformed body mass and log-transformed body length. It meets the assumptions of linearity (*r*=0.73) and independence [30]. Lizards were weighed using a hand-held Pesola scale to the nearest 0.1 g and SVL measured to the nearest 1 mm. LBSI has restricted use in this study as calibration curves for sex and other structural characteristics of *T. rugosa* (muscle, bone and fat mass) from different populations had not been determined.

### 3.5 Ectoparasite presence

The presence or absence of ticks for each individual was recorded by searching all surface areas of a lizard's body. All stages of both species of ticks, *Bothriocroton hydrosauri* (Acari: Ixodidae) and *Amblyomma* *limbatum* were highly visible especially with a hand lens. Tick load was measured as the number of ticks per individual and not grouped by species or tick life stage for this study

### 3.6 Statistical analyses

Multi-factorial analyses of variance using permutation tests, a RMA regression and Student *t*-tests were applied to health measures to test our claim that individuals of Severe sites will display ‘secondary effects’ of cultivation unlike Baseline individuals which should show pseudobasal values. Data were transformed where appropriate.

Differences in differential counts were examined using a multivariate, one-way nested Type I PERMANCOVA (analysis of covariance) [31–33], with LBSI or tick load as a covariate as the data were interdependent for each animal. Type I (sequential) analyses was chosen because the covariate(s) are fitted first and the sums of squares are no longer independent of one another. PERMANOVA analysis unlike traditional MANOVA was used for a number of reasons: (i) no explicit assumptions regarding the distributions of original variables especially multivariate normality and homogeneity of variances are made as tests are done by permutations, (ii) it is flexible and can be used with any dissimilarity or distance measure, (iii) it is intuitive as the test statistic is familiar as are measures of variation like ANOVAs, and (iv) it achieves a partitioning for any additive model. All analyses were conducted using Primer-E v6 PERMOVA add-on.

### 3.7 Post-mortem findings

During the study period, two sleepy lizards were found dead with injuries consistent with being run over by a vehicle in the Severe site. Representative tissues were collected from these animals, fixed in formalin, paraffin-embedded and 4 μm sections were routinely stained with haematoxylin and eosin and examined microscopically. A Prussian blue stain was done to assess liver iron concentrations.

## 4. Results

Data were collected from a total of 30 adults at the Baseline site and 75 adults and 14 young (juveniles and sub-adults) at the Severe sites [34]. Two animals in the Baseline site had exceptionally high total WBC counts of 39 184 and 74 667 cells μl^−1^, well above the range (4444–25 231 cells μl^−1^) of the other sleepy lizards sampled at Baseline site. We treated these animals as statistical outliers and they were not included in the subsequent analysis. To maintain orthogonal analyses between Baseline and Severe sites, 28 adult animals were randomly selected post hoc from the Severe site for statistical analysis.

### 4.1 Body condition

**Figure 2.**
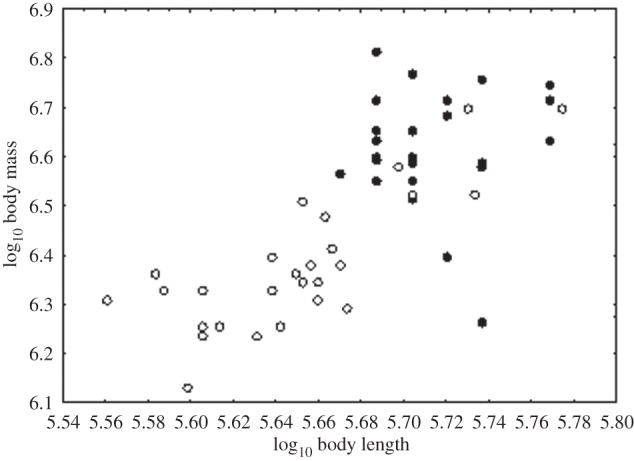
Relationship between log_10_ (body mass+0.1) and log_10_ (body length+0.1) for wild sleepy lizard *T. rugosa* in Baseline (solid circles) and Severe (open circles) sites of rangeland and croplands in southern Australia (*r*=0.73, RMA regression *F*_1,56_=20.94, *n*=56, *p*≤0.001).

Log-transformed body mass of adults was related to log-transformed body length (*r*=0.73, RMA regression *F*_1,54_=20.94, *n*=56, *p*≤0.001) (figure 2). Overall, Baseline adults were larger and heavier than those in the Severe sites whether the outliers were included or omitted in analyses (body length: Student *t*_length_=6.11, d.f.=54, *n*=56, *p*<0.0001; body mass: Student *t*_mass_=8.8, d.f.=54, *n*=58, *p*≤0.001). Twenty-one per cent (12) of animals weighed more than 800 g, 38% (21) between 640 and 775 g and 46% (23) between 460 and 600 g. Overall, LBSI was lower in the Severe sites than the Baseline site (table 1).

**Table 1.** Health indices summarized for individuals of the sleepy lizard *T. rugosa* at Baseline and Severe sites located in rangeland and cropland landscapes, respectively, of the Murray Mallee region in southern Australia.

health measures	Baseline mean±s.e. (CV%) (*n*=28)	Severe mean±s.e. (CV%) (*n*=28)
body condition		
LBSI	0.56±0.2 (142)	−0.53±0.1 (118)
body mass (g)	757.5±16.3 (1)	591.8±9.8 (9)
body length (mm)	301.8±1.4 (3)	284.1±1.7 (3)
number of ticks	13.1±6.8 (275)	6.5±2.2 (190)
differential counts		
heterophils (%)	54.7±2.1 (20)	49.9±1.8 (19)
lymphocytes (%)	15.8±1.7 (58)	29.2±1.3 (24)
monocytes (%)	6.6±1.5 (120)	10.7±1.4 (72)
other granulocytes (%)	21.8±1.7 (43)	10.2±1.1 (56)
H:L ratio	5.6±0.9 (92)	1.9±0.2 (50)
polychromasia (%)	1.7±0.4 (126)	13.2±1.5 (62)

Tick load had a disproportionate relationship on adult LBSI. At the Baseline site, tick load was notably higher and as a consequence log-transformed LBSI significantly decreased as log-transformed tick load increased (*r*=−0.52; *n*=28, *p*≤0.005). Conversely, adults at the Severe sites had a lower tick load which was not correlated with LBSI (*r*=−0.341; *n*=28, *p*≤0.076). Both LBSI and tick load were more variable in the Baseline than the Severe site, although these measures within each population were highly variable (table 1).

### 4.2 Haematology

**Figure 3.**
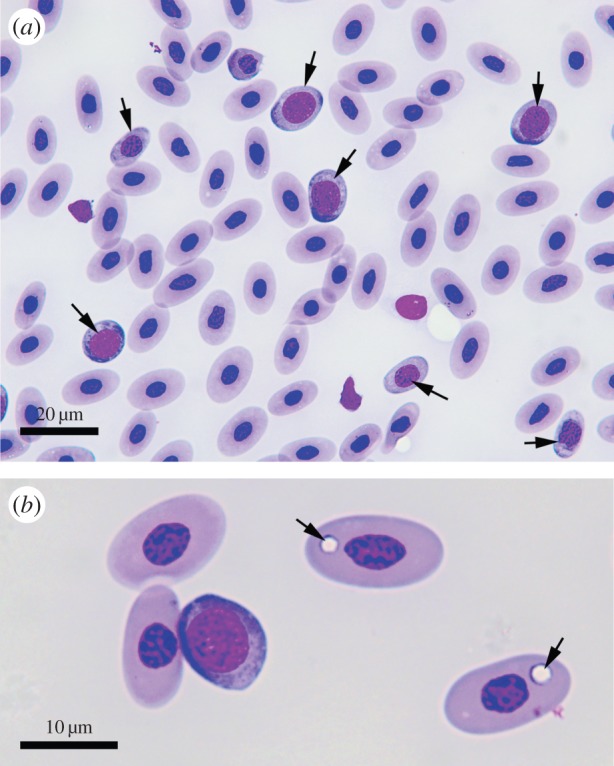
Photomicrographs of a Wrights Geimsa stained blood smear from an anaemic sleepy lizard from a Severe site demonstrating a marked increase in the number of polychromatophilic RBCs (arrows in figure 3*a*) and circular defects in the cytoplasm of mature RBCs (arrows in figure 3*b*).

Mean percentage of polychromatophilic RBCs were almost thirteen times higher in the Severe (13.2±1.5) than Baseline (1.7±0.4) sites (table 1). In this study, a very conservative cut-off of more than 10% polychromatic RBCs was used to define an animal with an abnormally high degree of polychromasia. Of 30 animals, 17 were recorded with values of more than 10% ranging between 11 and 34%. Cells in earlier stages of development than polychromatophilic RBCs were also commonly seen in the blood. All animals with high levels of polychromasia exhibited a normal sequence of the RBC maturation (figure 3*a*,*b*). Infrequently, mitotic figures were seen in RBCs in animals with elevated numbers of polychromatophilic RBCs. Mitotic figures were not seen in blood smears from animals in the Baseline sites. Mature cells stained uniformly and with normal intensity. Examination of mature RBCs revealed that the majority of the sleepy lizards from the Severe sites had at least some RBCs that had circular defects in their cytoplasm that did not stain, these were interpreted as areas where the cytoplasm was missing (figure 3*b*). These defects in the cytoplasm varied in diameter, but the diameter was never more than 20% of the length of the cell. Similar defects were not found in the RBCs collected from animals at the Baseline site. Blood parasites were not seen in any of the blood films examined.

In the Baseline sites, an additional three animals had WBC counts (not statistical outliers) that exceeded the high normal limit for other reptiles (15 000 cells μl^−1^) [20,35]. Although estimated WBC counts were not done on the samples from the Severe sites, a rough estimate of WBC density was made for each and none were judged to have values that exceeded the expected normal range. Average percentage of heterophils and other granulocytes was higher in the Baseline than Severe sites, whereas the mean percentage of monocytes and lymphocytes and heterophil to lymphocyte ratios (H:L ratios) were higher in the Severe sites (table 1). The variation for most of the haematological measures within the two sites was substantial but between sites the Baseline measures were consistently higher, suggesting the individuals in the Baseline site were responding to more environmental variation than the Severe sites.

**Figure 4.**
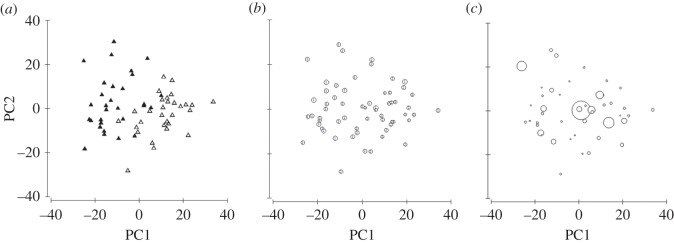
(*a*) Principal components (PC) ordination of differential count of WBCs with (*b*) log_10_ transformed LBSI and (*c*) log_10_ transformed tick load superimposed, showing separate groupings for LBSI and not for tick load on *T. rugosa* individuals. Baseline—filled or 1, Severe—open or 2, PC1—55.6% of total variation explained, PC2—26.7% of total variation explained. Baseline sites in (*a*) have mostly medium and large bubbles in (*b*), while Severe sites in (*a*) mostly have small bubbles in (*b*). Treatments not distinguishable in (*c*).

Excluding the differentials of two ‘outlier animals’, values of H:L ratio, monocytes and other granulocytes were square-root transformed due to right-hand skewness and all except heterophil differentials and polychromasia percentages were interdependent (electronic supplementary material, figure S1). Exploratory data analyses indicated that log-transformed LBSI and not tick load was structuring different haematology (figure 4). Despite there being a significant effect of LBSI structuring by haematology of lizards in the Baseline and Severe sites (PERMCOVA *pseudo F*_1,52_=10.08, *n*=56, *p*_(9948 permutations)_≤0.001), the differential haematology of adult lizards (measured by interactive percentages of heterophils, lymphocytes, monocytes, other granulocytes and H:L ratio) remained consistently different between Baseline and Severe sites (*pseudo F*_1,52_=12.60, *n*=56, *p*_(9960 permutations)_≤0.001) and this was not due to dispersion in distances from the centroids of the treatment levels, i.e. locational differences (*F*_1,54_=0.02, *n*=56, *p*≥0.8148). There was no interaction with LBSI and treatment (*pseudo F*_1,52_=1.34, *n*=56, *p*_(9960 permutations)_≥0.253). Principle component (PC)1, PC2 and PC3 principle component axes of the PERMCOVA explained 49, 28 and 14% (total explained *variation*=92%) which is also atypically high and a very consistent result. Differences among the differential counts were mostly weighted by lymphocyte percentage and no other haematological measures of Severe site animals (PC1). PC2 was largely weighted by heterophil percentage of Baseline site individuals and PC3 by polychromasia found in Severe site animals. H:L ratio explained less 1% in all ordinations.

### 4.3 Lizard packed cell volume and other potential factors influencing lizard health in the Severe sites

PCV was determined for 85 sleepy lizards in the Severe sites. The PCV ranged from 7 to 41% with an average of 25%. Forty-two lizards (49%) had PCVs of less than 25%, 16 of these (19% of the total) had PCVs of less than 20% and three animals had PCVs of less than 10%. PCV had no relationship to body condition, other haematological measures or elapsed sampling days since brumation (*p*≥0.05). Notably, there was no correlation between both arcsine transformed polychromasia (%) and H:L ratio (*r*=−0.01, *n*=75, *p*>0.34).

Habitat structural connectivity in the Severe sites had no detectable influence on adult and juvenile body condition and haematology (electronic supplementary material, box S2 and table S3), and are not discussed further.

### 4.4 Elapsed sampling time since brumation (CUMDAYS)

Sampling duration (CUMDAYS) was not correlated with any of the WBC health indices. However, per cent polychromasia of adults decreased significantly with CUMDAYS (Pearson *r*=−0.46, *n*=75, *p*=0.0002). All other differential indices had no consistent relationship with sampling duration.

### 4.5 Histological findings

Diffuse vacuolar degeneration (formation of non-lipid vacuoles in the cytoplasm) was present in the liver from one animal. This liver also contained a moderate degree of granular pigment in sinusoidal macrophages (phagocytic and antigen processing cells) and in zone 1 hepatocytes that was Prussian blue (iron) positive (not shown). No microscopic lesions were found in the second lizard. Although multiple sections of the digestive tract were examined in both animals, intestinal parasites were not seen.

## 5. Discussion

Declines in wildlife populations in agricultural areas can be caused by habitat alteration, exposure to agricultural chemicals and factors that may or may not be related to agricultural practices including infectious diseases. Identifying the factor or factors that may impact wildlife in agricultural environments can be challenging but is critical if processes are to be reversed. In this study, we use traditional field techniques (LBSI) for body condition score with less frequently used haematological techniques to assess the disease status and impacts of agricultural practices on sleepy lizards found in a cropping environment (Severe site) and a grazing environment (Baseline site) separated by a river. The experiment was also designed to determine whether differing environmental conditions within the Severe sites would have a greater or lesser impact on the LBSI and haematologic values.

The use of the LBSI and its components, body length and the animal's weight, as a measure of body condition demonstrated significant differences between the Severe and the Baseline animals, with the Severe site animals having a lower LBSI than those of the Baseline site. This finding appeared to confirm our original hypothesis that the fragmented and degraded Severe habitat was suboptimal and this suboptimal environment was reflected in the animals' condition. However, our data did not show any variation between sampling areas in the Severe site and the haematological results (discussed below), suggesting that widespread anaemia in animals in the Severe sites and not habitat itself may have resulted in the low LBSI, a conclusion that would not have been reached had haematology not been done.

Comparing physical characteristics, our findings also showed that phenotypically the adult sleepy lizards in the croplands, which are south of the Murray River barrier, are smaller in size (body length) than animals north of the river in the rangelands. This could suggest that extended geographical separation of these populations has resulted in genetic drift or selection resulting in animals in the Severe sites having a shorter body length. This hypothesis would be supported by a recent unpublished study that has found that the two populations come from different genetic lineages (Mike Gardner, in lit., 11 August 2014). Alternately, prolonged suboptimal conditions in the Severe sites, such as reduced food availability or protein malnutrition, might result in the animals not achieving their maximum growth potential. This alternative seems unlikely as food availability during the study in the Severe sites is less likely to be a factor owing to higher total rainfall in the southern SAMM.

Despite living in a fragmented environment and, in many instances, experiencing anaemia, H:L ratios were not influential in explaining differences for the animals in the Severe sites as compared with animals in the Baseline sites. Heterophil (reptiles, birds and some mammals) and neutrophil (mammal) to lymphocyte ratios have been used to assess stress in individual animals and populations of domestic and wild animals [21,35]. Typically, the percentage of heterophils increase and lymphocytes decrease resulting in an increased H:L ratio value with the release of glucocorticoid hormones as the result of acute and chronic stressors [35]. Interpretation of the H:L ratio, however, is fraught with complexities. Not all species respond the same way and changes in the H:L ratio may occur during the early stages of stress in some animals then return to more normal values in a matter of hours. Additionally, many factors, including concurrent inflammatory diseases and the impact of handling prior to sample collection can impact the H:L ratio in some circumstances making it a less useful measure of long-term environmental stress. Lastly, some species may respond to stress with a lowered H:L ratio and different sexes of the same species may respond differently to stress [36,37]. To date, there are only a very few studies that have looked at H:L ratios in reptiles in relationship to environmental stressors, and the results of a recent study suggest that the short handling times and immediate blood collection undertaken in our study meant that lymphocyte trafficking was unlikely or minimal at worst [28]. Further studies comparing blood or faecal corticosterone levels to H:L ratios in this species will be necessary before the value of H:L ratios can be determined.

Our finding of moderately to markedly elevated WBC counts in five (17%) of the sleepy lizards from the Baseline site negates our original assumption that samples from these lizards would be a reasonable representation of the basal range leucocytes for our species in the wild and illustrates the difficulty of linking leucocyte profiles to environmental condition for wild animals [2]. These values, however, suggest that a significant proportion of the animals in the Baseline population are experiencing a chronic inflammatory disease as significant increases in the absolute heterophil and monocyte counts often occur in these conditions [20]. Animals at this site were observed with ocular and nasal discharge at the time of the study by three of the authors. These observations suggest a possible infection with a *Mycoplasma* sp., a bacterium that has had significant impacts on wild reptile populations and thus this population requires an intensive disease investigation [38]. A correlation between WBC and observed signs in these animals would have been ideal, but was not recorded.

It would have been ideal to have used manual counting methods such as phloxine stains to determine the absolute total WBC counts, rather than depend on estimated counts from a blood smear [20]. This would have allowed accurate total WBC counts to have been determined for the sleepy lizards with anaemia which we could not determine using the estimate method from the blood smear. Technical errors at a commercial laboratory hired to do these absolute counts on the samples from the sleepy lizards in the Severe sites precluded use of these data.

Our study provides strong evidence that many of the sleepy lizards collected in the Severe sites were experiencing either a blood loss or RBC destructive (haemolytic) anaemia. The percentage of polychromatophilic RBCs in the lizards from these landscapes was significantly higher on average than the percentages in the less disturbed rangelands and very high values were seen in individual animals in the Severe sites, but not the rangelands, indicating that these animals were producing new RBCs at a greater rate in response to a decrease in RBC mass [35,39]. The PCV values obtained in this study also support the conclusion that the animals in the croplands were experiencing a decrease in RBC mass. PCV values were not obtained from the rangelands, but recent work with captive sleepy lizards suggests that the normal range for their PCV is between 25 and 35% (C. Moller 2012, unpublished data). This means that just under 50% of the lizards sampled in the croplands were at least mildly anaemic and 16% were moderately anaemic. Three animals had PCVs of 10% or less and these values were approaching those that would be considered lethal. We could not determine if the observed anaemia was the result of a single or ongoing insult because of conflicting findings. Anaemic lizards and lizards with elevated levels of polychromasia were identified at all times across the collection period which would be consistent with an ongoing insult and was not associated with brumation or seasonal changes [23]. However, because of a limited correlation between polychromasia and time, a single point exposure cannot be ruled out.

Blood loss was initially considered as a cause of anaemia. Ectoparasites, such as ticks that were found on these lizards and mosquitoes that would have been present in the environment can cause anaemia in their hosts. The tick burden found on animals from the Severe sites, however, was less than that found on the Baseline animals and although the tick load in the Baseline animals was correlated with a decrease in LCBI in this study and in a previous study [40], these animals had no evidence from their blood smear that they were anaemic. Mosquito counts in the area during the study period were also low, arguing against mosquitoes as a cause of blood loss (Waikarie–Loxton Council Annual Report 2010). Internal parasites can also cause blood loss. However, these were not identified in two lizards that were necropsied. The anticoagulant Pindone 2-(2,2-Dimethyl-1-oxopropyl)indane-1,3-dione (Animal Control Technologies, Sommerton, Victoria) is a rodenticide used to bait rabbits with poisoned oats in the croplands and sleepy lizards may eat baits containing it. This bait, however, is relatively expensive and is used locally around towns and would not have been used across the entire study area. Likewise, it is typically distributed in the summer (December to March) and not in the spring when this study was conducted.

Two lines of evidence support haemolysis and not chronic blood loss as the cause of the anaemia detected in these lizards. The first is the robust and orderly nature of the regenerative response seen in these lizards. This type of response is expected in both red cell destructive anaemia and acute blood loss. Chronic blood loss, which would have been more likely in these animals, given the presence of anaemia over the study period, would have resulted in a different haematological picture [20]. With chronic blood loss, the lizards would have become iron deficient and the polychromatophilic cells would have exhibited poor or variable cytoplasmic staining, as well as, variation in size and shape. Chronic haemolysis, however, does not result in iron loss and normal RBC regeneration would be expected as was seen in these lizards. The increased iron concentration in the liver of one of the necropsied lizards is consistent with the increase in iron turnover. Additionally, RBC membrane defects were observed in animals from the croplands but not the rangelands. These defects are probably the result of the increased presence of free radicals resulting in membrane damage and this in turn would have resulted in increased RBC destruction. These changes would not occur in animals with blood loss anaemia.

The causes of haemolytic anaemia are many. They can be immune-mediated where the body produces antibodies against an epitope on the surface of the cell. The epitope can be a normal cellular component or a viral, bacterial or parasitic protein, a drug or a toxin that is on the surface of the cell [41]. Blood parasites can induce haemolytic anaemia in reptiles but were not found in either of the study sites [22]. Toxins can also damage the RBC making it less flexible resulting in its premature destruction. Immune-mediated haemolytic anaemias are generally isolated incidents and it would be very unusual to see them occurring in animals across an entire ecosystem, therefore it is most likely that these animals were experiencing a haemolytic anaemia that was secondary to exposure to a toxin, possibly one that resulted in oxidative damage to the RBC membranes [41].

The lizards in the Severe sites were exposed to a variety of agrochemicals that could potentially act as toxins. Paraquat (*N*,*N*′-dimethyl-4,4′-bipyridinium dichloride) (Syngenta), a non-selective, contact-chemical weedicide, is used extensively in Severe sites on cropping farms and causes oxidative damage to animal tissue, particularly the lungs and kidneys, if ingested. Fox and rabbit baiting with the poison 1080 is also commonly used in this area and can be applied at any time during the year. Reptiles are generally considered to be less sensitive to the effects of 1080 as compared with mammals [42]. However, sleepy lizards from South Australia have been found to be more susceptible to the 1080 than sleepy lizards from Western Australia where this compound is found in the natural vegetation [43]. However, neither the frequency and timing (potentially anytime of the year) of baiting (A. Growdon, NRM Officer Mallee Coorong NRM Group, Department of Environment and Resource Management, South Australia) nor the use of Paraquat (April/March) has varied over the years. Additionally, lung and kidney lesions were not found in either of the two necropsied lizards making Paraquat toxicity less likely.

Locust and mouse plagues occurred during the study period resulting in the increased use of rodenticides and insecticides. Aerial spraying with Green Guard ULV (Becker Underwood Pty. Ltd) was done in November and December of 2010 east of the Baseline site. Green Guard contains fungal spores that kill locusts and a study on their impact on one reptile suggested that they were unlikely to be harmful [44]. Green Guard was not used in the Severe sites, instead ground spraying with the organophosphate chlorpyrifos (Lorsban500 EC, Dow Agrosciences) was done and local spraying by landholders with carbaryl, diazinon, fenitrothion, fipronil and various synthetic pyrethroids may have also occurred as indicated in South Australian government advisory fact sheets provided to farmers (http://www.pir.sa.gov.au (accessed March 2012)).

Both the organophosphate chlorpyrifos and fipronil could have played a role in the anaemia seen in the sleepy lizards in the Severe sites. Evidence of acute poisoning of reptiles has been observed in Africa following the application of chlorpyrifos to control the desert locust (*Schistocerca gregaria*) [13,44] and there is a report of a horse developing haemolytic anaemia after treatment with chlorpyrifos [45]. Additionally, evidence of increased oxidative stress has been found in a lizard (*Podarcis bocagei*) in Portugal exposed to ‘a cocktail’ of agricultural chemicals (including organophosphates, but not chlorpyrifos). Lizards in the exposed population from Portugal were more likely to exhibit hepatocyte vacuolation and this was also seen in one of the necropsied sleepy lizards [3,4]. In a controlled exposure trial, direct administration of fipronil and feeding of fipronil poisoned insects resulted in a high death rate in the fringe-toed lizard (*Acanthodactylus boskianus*) [44]. How the toxin killed these lizards was not investigated, but they did not exhibit the expected neurological signs and deaths were not immediate so other systems besides the neurological system may have been impacted.

On the other hand, the timing of the application of organophosphates and fipronil argues against them being the cause of the anaemia. Organophosphate application was only carried out from the beginning of October until the end of November. Anaemic animals and animals with polychromasia were found in September before the spraying occurred and therefore the only way that these chemicals could have been contributory would if the lizards were exposed to residual concentrations from the previous year.

Mouse baiting with wheat coated with zinc phosphide or wheat pellets containing zinc phosphide, increased significantly in 2010 and 2011 and these were distributed in the Severe sites and not the Baseline site. Most application would have occurred in March and April when the cereal crops were sown, but could have been applied in the winter and then again in the spring as well (K. Haebich, Team Leader, Rangelands and Rivers NRM Group, Department of Environment and Resource Management, South Australia). Sleepy lizards may have consumed these poisoned baits and it is also possible they could have consumed poisoned mice. Zinc phosphide is rapidly converted to phosphine gas when it is exposed to hydrochloric acid in the stomach. It causes cell death in many organs as the result of disruption of energy metabolism in the mitochondria. It affects RBCs by oxyhaemoglobin to methaemoglobin and thus could have resulted in the damaged RBCs seen in the sleepy lizards and the resultant haemolytic anaemia [46].

Sleepy lizards enter brumation soon after grain crops are sown. Studies on wound healing suggest that reptiles at temperatures below their prefered optimum temperature zone have a reduced metabolic rate and healing is delayed [47]. It would also be likely that animals in brumation would not be able to respond to a toxic insult during brumation and signs of exposure might persist until the following spring. We therefore consider it possible and even likely that sleepy lizards poisoned in the autumn may have still been anaemic in the spring as they emerge from brumation, and that additional application of zinc phosphide in the spring could have resulted in finding toxic changes during the entire study period. In the end, however, because of the exposure to multiple potentially toxic chemicals, the cause of the haemolytic anaemia seen in these sleepy lizards will require controlled exposure experiments before it can be determined.

The long-term impact of this haemolytic anaemia on the sleepy lizard population in the Severe sites is not known. Our data suggest that it resulted in reduction in body condition in many animals. However, given that animals with normal PCVs and blood smears were seen at the end of the study, it is possible that the sleepy lizards reduced their activities while recovering from the anaemia and because reptiles have relatively low metabolic rates were able to survive this insult.

In conclusion, this study shows the power of combining both physical and haematological measurements when assessing the health of reptiles across ecosystems. Using this combination of tools, we were able to demonstrate that sleepy lizards across a wide agricultural ecosystem were experiencing anaemia and a reduction in body condition that was likely to be the result of the application of one or more agrochemicals. We were also able to demonstrate that an infectious disease of potential ecological importance was present in the sleepy lizards in the rangeland site and that phenotypic differences could be found between sleepy lizards in the rangeland site and cropping site, possibly as the result of the physical barrier of a large river separating them. These findings also suggest that the sleepy lizard and possibly other reptile species may prove important sentinels for the impact of the use of agrochemicals on ecosystems and that more detailed and controlled studies need to be done on how these chemicals impact reptiles.

## Supplementary Material

Table S1. Summary of health indices for factors (habitat use type, structural connectivity, cumulative days since winter ‘hibernation’ and age class) recorded for the severe modification the sleepy lizard T. rugosa for the Murray Mallee region, South Australia. Box S1. Method and results of the influence of habitat complexity on the body condition and haematology of sleepy lizard Tiliqua rugosa Severe sites in the Murray Mallee region, southern Australia.

## Data accessibility

Haematological data are openly published at (doi:10.4227/05/537C5C5553F9E).
