# Supplementary material for: The use of body condition and haematology to detect widespread threatening processes in sleepy lizards (Tiliqua rugosa) in two agricultural environments
Source: R Soc Open Sci. 2014 Dec 24;1(4):140257. doi: 10.1098/rsos.140257 (PMC4448776; doi:10.1098/rsos.140257)
Supplement: Table S1. Summary of health indices for factors (habitat use type, structural connectivity, cumulative days since winter ‘hibernation’ and age class) recorded for the severe modification the sleepy lizard T. rugosa for the Murray Mallee region, South Australia. Box S1. Method and results of the infl [file rsos140257supp1.docx]

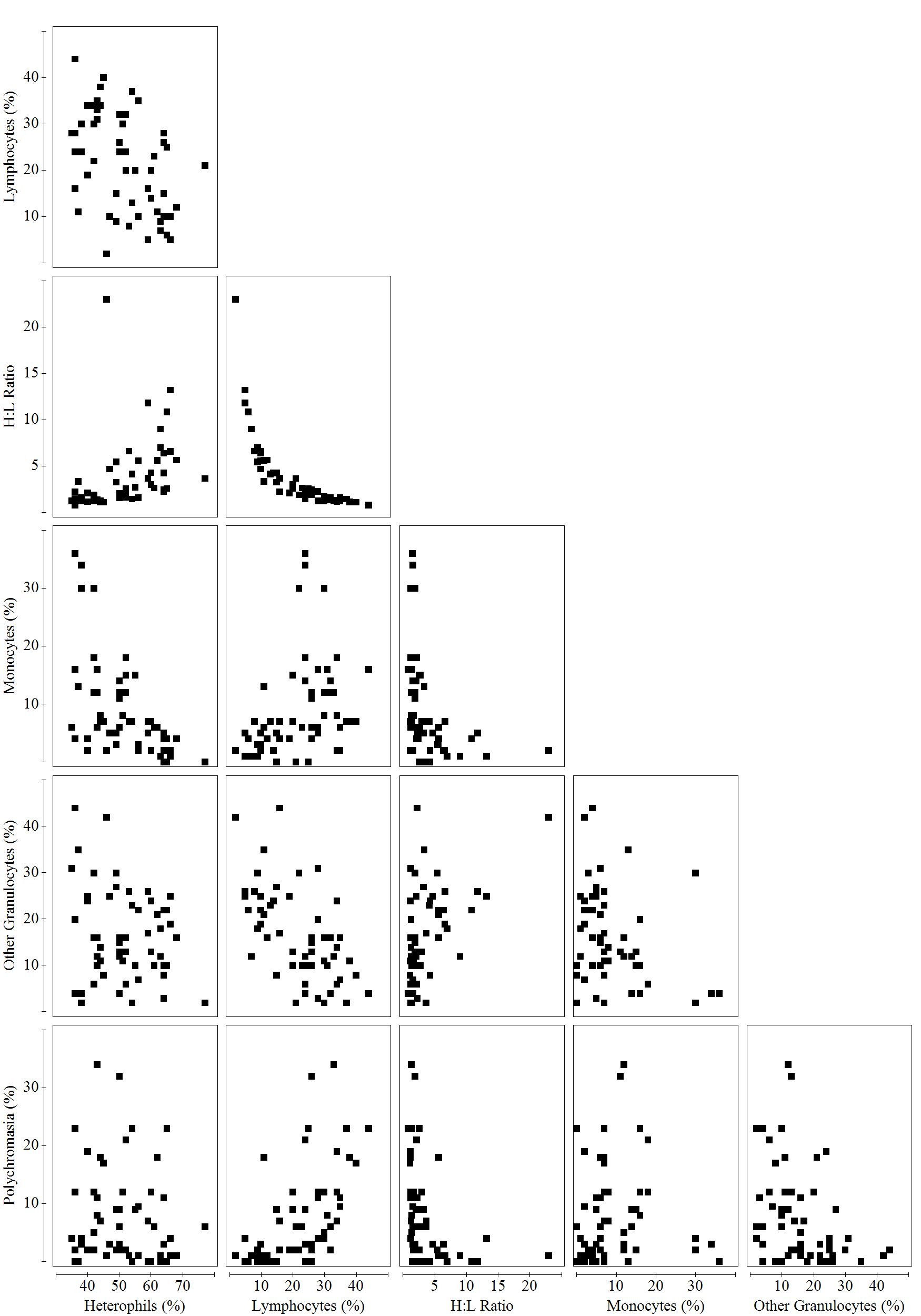


**Figure S1.** Differential counts of white blood cells of adult *Tiliqua rugosa* (*n* = 56) combined for both Baseline and Severe sites in the Murray Mallee region, southern Australia.

**Box S1.** Method and results of the influence of habitat complexity on the body condition and haematology of sleepy lizard *Tiliqua rugosa* Severe sites in the Murray Mallee region, southern Australia.

The influence of habitat complexity on lizard health in the cropland landscapes was studied using differences in two factors: (1) habitat type (fixed, 3 levels: remnant mallee woodland as small disconnected liner strips or patches with reduced habitat complexity, *n* = 27; revegetated saltbush, *n* = 28; crop/rested fields, *n* = 23) and (2) structural connectivity (fixed, 4 levels: low - < 63% connectedness, *n* = 24; medium – 63-88% connectedness, *n* = 25; high - > 88% but not connected, *n*= 19; connected to mallee woodland, *n* = 10). Both factors were nested within three replicated sites (LS1, LS2, LS3). These influences on body size and haematology (absolute counts and differentials) were examined using multivariate, one-way nested Type I PERMANOVA (analysis of variance) as the slope of each covariate was homogeneous (*p* > 0.05). We ran two separate analyses for habitat type and connectivity factors because the nature of historical vegetation clearance meant some levels of the structural connectivity were not represented in each of the levels of habitat type; some cells were empty with no replicates. All analyses were conducted using Primer-E® V6 PERMOVA® add-on. Statistical analyses indicated habitat complexity had no detectable influence on adult and juvenile body size and haematology as can be seen by the overlapping means and standard errors in the table below.

Habitat complexity had no consistent influence on body condition, leucocytes and days since brumation (Table S3)

**Table S1.** Summary of health indices for factors (habitat use type, structural connectivity, cumulative days since winter ‘hibernation’ and age class) recorded for the severe modification the sleepy lizard *T. rugosa* for the Murray Mallee region, South Australia.

|  | adult habitat use type | | | adult structural connectivity class | | | | age class | |
| --- | --- | --- | --- | --- | --- | --- | --- | --- | --- |
|  | native mallee remnant  mean±SE  (*n* = 27) | revegetated  saltbush  mean±SE  (*n* = 28) | cereal /rested field  mean±SE  (*n* = 23) | low  mean±SE  (*n* = 24) | medium  mean±SE  (*n* = 25) | high  mean±SE  (*n* = 19) | connected  mean±SE  (*n* =10) | juvenile/  subadult  mean±SE  (*n* = 14) | adult  mean±SE  (n = 75) |
| body condition |  |  |  |  |  |  |  |  |  |
| LBSI | -0.52±0.2 | -0.21±0.2 | -0.07±0.1 | -0.34±0.2 | -0.33±0.1 | -0.15±0.2 | -0.15±0.2 | 0.26±0.1 | 7.3±0.2 |
| body mass (g) | 460.78±38.4 | 519.2±35.1 | 538.9±28.2 | 510.0±40.6 | 483.0±32.5 | 524.74±40.3 | 518.7±50.9 | 225.4±16.5 | 6.5±2.2 |
| body length (mm) | 257.0±9.0 | 269.1±6.7 | 270.9±6.7 | 267.2±9.2 | 262.72±7.9 | 266.3±7.8 | 267.1±11.3 | 204.5±4.8 |  |
| number of ticks | 1.4±0.5 | 1.2±0.4 | 1.2±0.4 | 1.4±0.6 | 1.2±0.1 | 1.1±0.2 | 1.6±0.1 | 1.8±0.6 |  |
| differential count |  |  |  |  |  |  |  |  |  |
| heterophils (%) | 53.6±2.0 | 52.9±1.8 | 49.6±2.1 | 53.3±2.0 | 52.4±2.1 | 50.1±2.2 | 52.6±3.6 | 55.3±2.8 | 51.8±1.0 |
| lymphocytes (%) | 29.1±1.3 | 28.9±1.3 | 29.4±1.2 | 28.7±1.3 | 29.7±1.5 | 28.9±1.4 | 28.9±1.8 | 25.6±2.3 | 29.3±0.7 |
| monocytes (%) | 7.2±1.0 | 8.1±1.1 | 10.9±1.8 | 8.3±1.2 | 7.9±1.5 | 10.5±1.6 | 8.1±2.0 | 8.4±2.0 | 8.8±0.7 |
| other granulocytes (%) | 10.0±1.1 | 10.0±2.0 | 10.1±1.5 | 9.5±1.4 | 10.0±1.4 | 10.5±1.4 | 10.4±2.3 | 2.4±0.5 | 3.1±0.3 |
| H:L ratio | 2.0±0.1 | 2.0±0.2 | 1.8±0.1 | 2.0±0.1 | 2.0±0.2 | 1.8±0.2 | 1.9±0.2 | 2.6±0.4 | 1.9±0.1 |
| polychromasia (%) | 13.1±2.0 | 11.4±1.7 | 15.9±2.0 | 13.2±2.4 | 15.0±1.9 | 13.4±2.2 | 9.3±2.6 | 12.9±1.8 | 13.5±1.1 |
| CUMDAYS | 36.2±1.5 | 36.0±2.3 | 36.9±1.9 | 35.5±2.1 | 36.6±1.6 | 38.5±2.0 | 33.9±3.7 | 31.5±2.7 | 37.2±1.1 |
